# Comparative immunohistochemical analysis of Beclin-1 & MDM-2 in benign & malignant ameloblastomas

**DOI:** 10.1186/2051-1426-3-S2-P101

**Published:** 2015-11-04

**Authors:** MA Elbarrawy

**Affiliations:** 1 Alexandria University Egypt, Alexandria, Egypt

## Background

Ameloblastoma is the most frequently encountered neoplasm arising from the odontogenic epithelium. Beclin-1 protein plays a critical role in autophagy as a tumor suppressor gene. Whereas, the Murine Double Minute 2 (MDM-2) is a cellular proto-oncogene capable, if amplified, of causing tumor-genesis. The expression & prognostic significance of both genes are largely unexplored, yet, in this neoplasia. Therefore, the present investigation aimed to assess their possible biological role in ameloblastomas.

## Methods

This study was done among 35 studied cases: 29 cases of benign ameloblastomas, and 6 cases of ameloblastic carcinomas. Labeled Streptavidin Biotin (LSAB + Dako) immunohistochemical method, utilizing monoclonal antibodies for Beclin-1 & MDM-2 genes, was used.

## Results

Most of the benign ameloblastomas, 25 out of 29 cases (86%), showed intense total cell positivity for the Beclin-1, while, the ameloblastic carcinomas revealed mild (3 out of 6 cases, 50%) to negative expression (3 cases: 50 %). Inversely, the MDM-2 oncoprotein demonstrated intense brown total cell reactivity in amelobastic carcinoma (5 out of 6 cases, 83% positivity) & loss of the reaction (21 cases: 72%) to mild brown stain (8 cases:28%) in benign ameloblastoma. These findings were statistically significant.

## Conclusion

Based from these findings, one could conclude that, MDM-2 could be a specific marker to identify the proliferative activity, tumor aggressiveness & directly proportional with the degree of malignancy. In contrast, the high Beclin-1 expression could be a good indicator of prognosis in ameloblastomas. Hence, an overall comparison, both studied genes may be very promising molecular prognostic biomarkers.

